# Liquid neural networks for adaptive DDoS detection in IoT and SDN environments

**DOI:** 10.1038/s41598-026-67535-5

**Published:** 2026-08-19

**Authors:** Mubashar Raza, Muhammad Awais Sattar, Zarmina Jahangir, Mustaeen Billah

**Affiliations:** 1 Department of Computer Science, COMSATS University, Sahiwal Campus, Islamabad, Pakistan; 2 Automatic Control, Department of Computer Science, Electrical and Space Engineering, Luleå University of Technology, Luleå, Sweden; 3 Riphah School of Computing and Innovation, Riphah International University, Lahore Campus, Lahore, Pakistan; 4 Department of Business, Robert H. Smith School of Business, College Park, MD USA

**Keywords:** Liquid Neural Networks (LNNs), Distributed Denial of Service (DDoS), Temporal Adaptation, Internet of Things (IoT), Software-Defined Networks (SDN), Engineering, Mathematics and computing

## Abstract

DDoS attacks continue to threaten Internet of Things (IoT) and Software Defined Networking (SDN) systems, where network traffic is highly dynamic and changes rapidly over time. Most machine learning and deep learning-based detection models assume stable traffic distributions and therefore struggle when attackers vary packet rates, flow intervals, and traffic intensities to avoid detection. Liquid Neural Networks (LNNs) offer a more suitable approach because they operate in continuous time and can adjust their internal states in response to time varying inputs. This study explores the use of LNNs for adaptive DDoS detection in IoT and SDN environments and evaluates their ability to model evolving and adversarial traffic patterns. The proposed framework uses three stacked liquid time constant layers combined with a multi head attention mechanism to learn both short term and long-term temporal dependencies in network flows. Three recent datasets, CICIoT2023, SNT SDN2025, and IoT23, were preprocessed through cleaning, normalization, and consistent feature scaling. The model was trained with the Adam optimizer and evaluated using accuracy, precision, recall, false positive rate, and false negative rate. Robustness was assessed using a multiple input multiple output adversarial testing approach based on the Momentum Iterative Method, which generates subtle perturbations to simulate real evasion attempts. The results show that the LNN based model achieves high performance on all datasets, with a maximum accuracy of 99.96% and significantly lower false positive and false negative rates compared with all baseline models including multilayer perceptron (MLP), LSTM, GRU, and CNN-LSTM. Under adversarial conditions, the LNN model retains more than 90% accuracy, while the strongest baseline CNN-LSTM drops to a maximum of 84.95%. These findings indicate that LNNs effectively capture the temporal variability present in DDoS traffic and generalize well across diverse attack scenarios. Overall, the results demonstrate that continuous time adaptive computation enables LNNs to provide robust, efficient, and temporally aware DDoS detection suitable for practical deployment in IoT and SDN environments.

## Introduction

DDoS attacks cause severe damage to traditional and dynamic network environments^1^. DDoS attacks can impact businesses financially and damage their reputation^2–4^. IoT and SDN networks have large number of devices with diverse nature and limited computational resources which increase chances of DDoS attacks^5–7^. Temporal adaptation is an ability of malicious actors to modify and evolve their attack patterns over time in order to avoid intrusion detection systems (IDS)^8^. In case of DDoS attacks, attackers rarely maintain a fixed behavior and approach. Instead, they continuously adjust factors such as packet rates, flow durations, traffic intervals, attack sources and attack intensity to mimic normal traffic patterns and to bypass IDS^9,10^. This temporal variation makes attacks more stealthy and harder to distinguish from legitimate traffic^11^. SDN and IoT traffic changes and evolves over time which creates difficulty for traditional machine learning (ML) and deep learning-based models to detect DDoS attacks^12^.

ML and DL models are widely used for DDoS detection in SDN and IoT environments, but most of them typically assume that statistical properties of traffic remain relatively stable over time^13^. Temporal adaptation by attackers breaks this assumption, causing a problems^14^. Beyond temporal adaptation of real DDoS traffic patterns, detection models also face a second distinct threat in the form of adversarial attacks, where an attacker deliberately crafts input features to cause misclassification, an aspect that most existing DDoS detection studies do not address. A model trained on past data may fail to recognize new or slightly modified attack patterns, leading to increased false negatives. Similarly, legitimate SDN and IoT traffic that evolves over time may be misclassified as malicious, increasing false positives. Result is that static ML models quickly become outdated in dynamic environments, losing accuracy and reliability^15^. It creates a vital challenge which is DDoS detection systems should not only identify current threats as well as continuously adapt to developing traffic behaviors formed by attackers and IoT networks itself^16,17^. LNNs are advanced and modern type of neural networks. They are designed to handle time varying data which make suitable for detection systems in SDN and IoT environments^18–20^. For DDoS detection, this means LNNs can learn shifting characteristics of attack traffic as attackers adapt their strategies over time, addressing problems that weakens conventional ML and DL models^21^.

This study presents an advanced DDoS detection system based on LNNs. By utilizing dynamic computation capability of LNNs for varying traffic of DDoS, proposed model provides an advanced and practical solution for security of IoT and SDN system. Proposed model was comprehensively evaluated using latest and modern datasets specifically created for IoT and SDN environments, including CICIoT2023^22^, SNT-SDN2025^23^, and IoT23^24^. Experimental results demonstrate that LNN-based framework achieves consistently high detection accuracy, precision, and recall. Furthermore, results highlight capability of LNNs to provide temporal adaptation in volatile traffic conditions, outperforming traditional ML and DL models in both efficiency and effectiveness. These findings confirm suitability of LNNs for real-time intrusion detection in dynamic network environments.

### Literature review

SDN provides flexibility and dynamic approach towards network management through data plane, control plane, application programming interfaces (APIs) and centralized control management^16,25^. Enterprises use SDN to deploy applications faster with low cost^26^. Central dependency of network results in single point of failure^27^. SDN is prone to DDoS attacks due to its centralized nature^28^. In a DDoS attack attackers try to overwhelm network or server by sending a massive amount of fake traffic^28^. DDoS attacks can be categorized in many categories depending upon their nature^27^. Although all of the DDoS attacks are harmful for SDN but control plane saturation is usually most dangerous for SDN because if controller is overwhelmed, whole network loses its “brain” ^25,29^. Control plane saturation attacks occur when an attacker floods network with traffic designed to create a huge number of new flows, forcing switches to constantly query controller for instructions^16^. Each new, unmatched flow triggers a packet-in message to controller, this quickly consumes its CPU, memory, and bandwidth. As controller is central decision-maker, overwhelming it leads to severe slowdowns, dropped packets, or even a complete failure^27^.

IoT devices are connected to internet, collect, share, and act on data^3,30^. Each IoT device has sensors to gather data, processors to interpret it, and communication modules to send or receive information over network^3^. It delivers insightful details by analyzing the data from the multiple sources, that can help businesses to optimize operations, predict issues, and decrease downtime^31^. IoT devices have limited computational power^5^. Privacy is another concern, as IoT devices often collect sensitive data that could be exposed. IoT networks are also prone to DDoS attacks because many connected devices have weak security, and can be easily hijacked^4^. Continuous connectivity of IoT devices and their dependence on internet make them more exposed and harder to secure^,24,31–33^. IoT devices themselves can be compromised and turned into a botnet that launches DDoS attacks against external targets^30^. IoT networks can also be direct targets of DDoS attacks^4^.

Yousif et al., ^34^ proposed a one dimensional convolutional neural networks (1D-CNNs) model which uses a stacked convolution technique and combines mininet and Ryu controller. They tuned seven hyperparameter of trained 1D-CNNs model using non-dominated sorting genetic algorithm (NSGA-II). They proposed a model and framework for DDoS detection in SDN networks. They claimed that their proposed model is better than traditional support vector machine (SVM), logistic regression (LR), k-nearest neighbor (KNN), and random forest (RF) models. They claimed that their proposed model is highly optimized and accurate. Noe et al.,^35^ presented a SDN based architecture which is modular and flexible to detect transport and application layer based DDoS attacks using various ML and DL techniques including K-NN, RF, SVM, multilayer perceptron (MLP), gated recurrent units (GRU), CNN and long short-term memory (LSTM) neural network. Authors evaluated and related these methods and approaches for DoS and DDoS attacks detection in SDN based systems and networks. They also said that they utilized a simulated environment with the help of open network operating system SDN controller and mininet emulator.

Daniel et al.,^36^ presented an anomaly detection system to mitigate and detect DDoS attacks in SDN using unsupervised generative adversarial neural networks (GANs) along gated recurrent units (GRU). They also proposed a mitigation algorithm for DDoS attacks on SDN. They claimed that their proposed research work is highly efficient against DDoS attacks and it can detect and mitigate attacks on SDN environments with accuracy. Mah-Rukh et al., ^33^ introduced a dual stage defensive framework based on SDN for IoT networks. They presented framework to mitigate DDoS attacks on IoT systems. They claimed that to mitigate these attacks, detected attacks are classified at network controller, enabling timely updates to data plane defensive rules. They evaluated their framework on CICIoT2023 dataset. They said they deployed separate detectors on data plane for DDoS attacks which reduced false alarms by 58%. They said as a part of their framework, CNN based classifier achieved 99% accuracy. They simulated evaluation environment using mininet emulator. They said their framework can also handle web-based attacks in same fashion. They said their framework reduced link load by 99.8%.

Faris et al., ^37^ presented a solution to secure IoT devices against DDoS attacks. Their proposed model consists of two components, a server detector and an IoT node detector. They used LSTM for training and prediction. They said that they also tested their proposed model using random sequences and found that their model was efficiently working. They said their proposed model gave promising results during evaluation. Yonas et al., ^6^ proposed a multistage adversarial attack defense framework to secure online DDoS attacks detection systems from the adversarial attack in IoT systems. Their proposed model consists three defense layers, one layer consists of resilient adversarial detector and purification, that detects adversarial attacks aiming online DDoS attack detection system. Second layer consists of a multi classifier, which increase complexity for a hacker to replicate DDoS attack detection module. Third layer consists of a multi-armed bandit along Thompson sampling, that dynamically selects optimal classifier for each incoming traffic request.

LNNs are based liquid time constant networks (LTCs)^38–40^. LNNs was first introduced by Hasani et al., ^38^. LNNs can adapt to any dataset regardless of its complexity. Term “liquid” characterizes network’s adaptive time-constant, which allows it to flexibly process temporal information^39^. Traditional deep learning models process time in fixed, predetermined intervals^20^. In a LNN, however, time intervals are dynamic they adapt using system’s current state because time-constant is attached with hidden state^38^. LNNs are extremely flexible or liquid which enable them to handle unpredictable changes in data^39^. LTCs are practical application of LNNs. Architecture of LTCs is similar to linear first order dynamical systems^39^. Neurons in LNNs are linear and connected through non-linear gates^39^. Traditional feed forward neural networks use layers of neurons and connections among them to perform nonlinear calculations to learn patterns in training data^20^. Traditional feed forward neural networks use non-linear function at each neuron to find and calculate output whereas LTCs utilize linear model, for example, first order ordinary differential equations (ODEs)^39^.

ODEs are simpler and easy to calculate outputs^38^. LTCs manage and regulate these linear models with the help of non-linear functions or gates^39^. Differential equations (DEs) define how things change with respect to time and are commonly used in applied engineering and physics. In simple words ODEs are simple DEs^39^. In their research work Hasani et al., ^39^ claimed that LNNs can perform 5% to 70% better as compared to traditional deep learning and other models. They also said that LNNs are lightweight as they use less parameters as compared to other advance models^39^. Unlike traditional DL models, which often rely on static data and struggle when attackers modify their methods, LNNs maintain a dynamic internal state that evolves with incoming data^20^. This temporal adaptability allows them to capture shifting attack behaviors, such as variations in packet rates, distribution, and sources, without requiring frequent retraining. The unique architecture permits them to regularly update internal states based on incoming traffic and its data, providing them with more effectiveness in detecting fast varying and unpredictable attacks behaviors for example, DDoS and DoS attacks. This adaptability provides LNNs to achieve high accuracy with a fewer number of parameters, and improved generalization, that is important in dynamic networks and their environments^40^. A review of existing LNN applications reveals that prior work has focused primarily on domains such as robotic navigation, time-series forecasting, graph neural networks, and IoT device classification. Reference^21^ represents the closest related work, applying LNNs to IoT device identification rather than intrusion or attack detection.

A systematic survey of LNN-based research from 2021 (when LTCs were introduced) through 2026 confirms that prior work has explored LNNs across a range of domains, none of which includes network security or DDoS detection. In robotics and autonomous systems, Chahine et al. ^20^ applied LNNs to flight navigation, and the Liquid-Graph Time-Constant Network (LGTC) was developed for multi-agent control Marino et al., ^41^. In time-series anomaly detection, LTC-AE autoencoders were proposed for generic streaming data Riya et al., ^42^ but these were not applied to network traffic or security contexts. In the IoT domain, Kumar et al., ^21^ applied LNNs to IoT device identification, which is a classification task fundamentally different from intrusion or attack detection. In graph neural networks, Zhang et al., ^19^ proposed an LNN-inspired graph framework for sequential recommendations. In wireless communications, Wang et al., ^18^ used LNNs for robust beamforming. The only work approaching a security-adjacent application of continuous-time recurrent networks is a 2025 study applying LTC and CFC networks to misbehavior detection in Internet of Vehicles (IoV) Gong et al., ^43^, which addresses internal vehicular network anomalies rather than external DDoS traffic classification. None of these works, individually or collectively, applies LNNs to DDoS detection, SDN security, IoT intrusion detection, or any network-level attack classification task.

A recent systematic review by Polat et al. ^44^ supervised and deep learning techniques for DDoS detection in SDN-based networks, extending the analysis across IoT, SCADA, 5G/mobile, and VANET domains. Their taxonomy of deep learning approaches, spanning CNN, LSTM, GRU, MLP, and ensemble/hybrid models, does not include liquid neural networks or liquid time-constant architectures, and their review does not evaluate adversarial robustness, reinforcing the novelty gap identified in this work. Several complementary approaches to DDoS and intrusion detection have been proposed in related domains. Fuzzy logic-based detection systems have been explored for anomaly and DDoS detection, demonstrating the effectiveness of non-crisp classification boundaries for handling uncertain network traffic patterns^45^. Attention-based hybrid architectures combining bidirectional LSTM and CNN have been applied to SDN and IoT DDoS detection, achieving strong results through sequential feature extraction and attention weighting^46^. Machine learning-based frameworks using classical algorithms have established foundational benchmarks for DDoS detection in networked environments^47^. More recently, hybrid data balancing strategies combined with probabilistic classification models have been proposed to address class imbalance challenges in IoT intrusion detection, highlighting the importance of robust training data preparation for reliable detection performance^48^.

A systematic search was conducted across IEEE Xplore, ACM Digital Library, and arXiv using keywords including ‘liquid neural network’, ‘liquid time-constant’, and ‘LTC network’, combined with ‘DDoS’, ‘intrusion detection’, ‘network security’, and ‘anomaly detection’, covering publications from 2021 through early 2026. No relevant results were returned. No prior study has applied LNNs to DDoS detection, network intrusion detection, or any network security classification task, establishing a clear novelty gap that this work addresses.

### Limitations and drawbacks of existing studies

Several approaches have been presented and proposed for DDoS detection in SDN and IoT networks, important limitations persist^1,8,10,25,27,34,35,49,50^. Most existing methods rely on traditional DL or ML models^2,33,35,36^. These models are typically trained on fixed datasets that do not reflect diversity of IoT devices, traffic behaviors, and attack strategies in real networks, limiting their generalization across deployments. A further limitation is lack of temporal adaptation. Current models do not update their internal parameters in response to incoming traffic^11,31,35^. As attack strategies evolve quickly, models without temporal awareness often detect only known attacks and fail to recognize new or stealthy variants. Detection of them requires costly and time consuming retraining, that reduces their practicality in dynamic SDN and IoT environments^26^. Present studies often ignore issues such as scalability, adversarial resilience, and temporal adaptation^27,34,36^. These gaps collectively make current approaches less effective against large-scale, dynamic DDoS attacks in dynamic network environments. These limitations highlight need for detection models that are adaptable over time, and explainable, while maintaining reliable performance in SDN and IoT environments. Table 1 summarizes limitations of existing studies.

### Motivation

Increasing reliance on SDN and IoT has been made these environments gradually attractive targets for DDoS and DoS attacks^16,25,49^. Existing approaches rely on static models requiring frequent retraining, which restricts their generalization to real-world conditions. Absence of temporal adaptation further limits their ability to recognize evolving attacks. Beyond these performance gaps, many studies do not address practical challenges such as temporal adaptation, scalability, adversarial resilience, and explainability^8,12,31,49^. Adversarial robustness is needed to withstand sophisticated attack strategies, and explainability is critical for ensuring accountability. LNNs emerge as a strong candidate to address these challenges. Their efficient and compact architecture supports deployment in SDN and IoT environments, while their continuous time dynamics permit temporal adaptation to shifting traffic patterns. Unlike conventional DL models, LNNs provide more transparent internal states, enhancing interpretability and trust. These properties position LNNs as a promising basis for building DDoS detection systems.

### Main contributions

To the best of our knowledge, this paper is the first to use LNNs for adaptive DDoS detection in IoT and SDN networks. LNNs work in continuous time varying setting and can change their internal state quickly, which helps them follow swift shifts in network traffic. This makes them a good fit for IoT and SDN environments, where conditions often change fast and DDoS attacks can appear in different forms. In this paper we use these properties to build a model that can adjust to new traffic patterns and react more reliably during DDoS attacks. We reviewed research from 2020 to 2026, starting from when LNNs were introduced, and we did not find any earlier studies that used them for adaptive DDoS detection in either IoT, SDN, or both together. More broadly, to the best of our knowledge, no prior study has applied LNNs to any form of network intrusion detection system (NIDS), making this the first work to establish LNNs as a viable architecture for network security applications.

Significant contributions of this research can be summarized as follow:

- LNNs based model is proposed for detecting DDoS attacks in SDN and IoT environments, addressing potential gaps of existing studies.
- Proposed DDoS detection framework leverages temporal adaptability of LNNs to capture evolving traffic dynamics. This allows model to detect both known and developing DDoS attacks patterns without requiring costly retraining.
- Extensive level experiments are carried out using latest benchmark and recent DDoS datasets. Results show that proposed approach achieves high detection accuracy, lowest FPR and FNR rates making it appropriate for deployment in IoT and SDN environments.
- Robustness and generalization capability of proposed model is evaluated against adversarial attacks using a multiple input multiple output (MIMO) based adversarial framework to simulate evasion attempts in IoT and SDN environments.
- Integration of a multi-head attention layer improves feature representation by focusing on critical temporal dependencies within network flows, improving both interpretability and detection accuracy.
- An ablation study is conducted to quantify the individual contribution of each architectural component of the proposed model, including the LTC layers, multi-head attention mechanism, batch normalization, and dropout regularization, confirming the necessity of each design choice.
- This study shows that LNNs provide more interpretable internal states compared to conventional DL and ML models. This improves trust and facilitates practical adoption in security sensitive SDN and IoT applications.

**Table 1:** Limitations of existing research works.

Studies	Year	Contributions	Limitations
^1^	2023	They proposed an SDN based framework for the detection of slow rate DDoS.	This study does not address temporal adaptation and adversarial resilience. They evaluated the model using only one dataset.
^2^	2024	This study presented an LSTM-based approach for DDoS detection.	They evaluated the model using only one dataset. The model’s resilience to adversarial DDoS attacks is not addressed.
^11^	2024	The study proposes a ML based hybrid framework for DDoS detection and mitigation in SDN-enabled smart home networks.	The framework relies on a limited set of ML models. The system’s temporal adaptability and adversarial resilience are not evaluated.
^49^	2024	The study presents a ML-based DDoS detection framework for SDN-enabled IoT environments. It establishes a testbed environment for simulating DDoS attack traffic.	Framework relies on traditional ML models that could not handle dynamic IoT traffic more effectively. Study does not examine temporal adaptation and adversarial resilience. Evaluation is limited to simulated traffic within a controlled testbed.
^25^	2021	They proposed RNN-LSTM model for detecting and mitigating DDoS attacks. They also performed the comparative analysis of DL and ML models.	Accuracy of proposed model remains good but not impressive 89.63%. Study lacks temporal adaptation and does not explore adversarial robustness. Evaluation of proposed model is limited.
^33^	2025	This study introduces dual stage defensive model designed to detect and mitigate DDoS and web based attacks in IoT environments. Framework integrates a lightweight decision tree model and a CNN classifier.	Framework is evaluated on a single dataset that can limit its generalizability to other IoT networks environment. Model lacks temporal adaptation and adaptability to evolving attack patterns.
^34^	2023	The study presents an optimized DDoS detection framework that integrates Mininet, the Ryu controller, and 1D-CNN.	The use of a single DDoS dataset limits the assessment of generalization. While NSGA-II improves accuracy, it also introduces computational overhead.
^35^	2021	The study presents a flexible architecture for detecting DDoS attacks using a combination of ML and DL techniques.	The study does not discuss temporal adaptability and assessment of adversarial resilience. The integration overhead of multiple ML and DL models is not discussed.

## Methodology

In the IoT and SDN environment DDoS attacks are particularly disruptive because they can overwhelm SDN controller or resource-constrained IoT devices^10,51^. Detecting such attacks is essential for maintaining service availability and securing network operations. DDoS detection can be defined as a classification problem in which network traffic is analyzed to determine whether it is benign or malicious^27^. Traffic monitoring and attack detection system then predicts whether observed traffic corresponds to normal behavior or an ongoing attack on devices. This kind of problem is challenging and vital for three key reasons. First of all, traffic patterns and attack strategies evolve continuously, requiring models that can adapt over time. Second, SDN and IoT environments are resource-constrained and latency-sensitive, which limits applicability of complex detection mechanisms that require heavy retraining. Third, explainability is important for practical deployment. Central goal of this work is to design a detection approach which not only achieves high and better accuracy, but also adapts to evolving traffic dynamics while meeting requirements of SDN and IoT environments.

Let incoming network traffic at time step *t* be represented as a feature vector and n denotes the total number of extracted traffic features per sample as showed in Eq. 1:

1 $$\:{X}_{t}=\{{x}_{t1},{x}_{t2},{x}_{t3},{x}_{t4}\dots\:,{x}_{tn}\}$$

where $\:{x}_{ti}$ denotes *i-th* extracted traffic feature (e.g., packet rate, flow size, protocol distribution) at time *t*. Each traffic sample is associated with a ground truth label mentioned in Eq. 2:

2 $$\:{y}_{t}\in\:\left\{\mathrm{0,1}\right\}$$

where $\:{y}_{t}=0$ represents benign or normal traffic and $\:{y}_{t}=1$ denotes malicious (DDoS) traffic. The objective of DDoS detection system is to learn a mapping function:

3 $$\:{f}_{\theta\:}:{X}_{t}\to\:{y}_{t}$$

Parameterized by *θ*, such that predicted label $\:{y}_{t}={f}_{\theta\:}\left({X}_{t}\right)$ correctly identifies benign or attack traffic. Unlike static classification tasks, DDoS detection in SDN and IoT presents unique challenges including temporal adaptation, scalability, and explainability. Formally, goal is to minimize classification loss over streaming traffic data while satisfying constraints.

Primary objective of this research work is to detect DDoS attacks in dynamic network environments using LNNs. Traditional ML and deep learning approaches rely on static traffic patterns, which limits their ability to adapt when network conditions shift rapidly. LNNs, by contrast, introduce time-dependent neuron dynamics that allow model to continuously adapt to changing input distributions and patterns. Problem is consequently formulated as a supervised classification task: given a set of network traffic features at a given time, model must classify flow as either benign or malicious (DDoS). In evaluation, this study simulates dynamic environments by combining heterogeneous datasets and varying traffic intensity across time. This design allows proposed model to be tested under conditions that better reflect real-world deployment, where attack vectors and legitimate traffic evolve continuously.

Proposed model was trained and tested separately on three datasets to assess its generalization capability across diverse DDoS attacks, feature sets, and traffic conditions^22–24^.

MLP was selected as the baseline model because it represents the class of static feedforward architectures that are widely used in DDoS detection literature, providing a reproducible and meaningful point of comparison. More importantly, MLP lacks any temporal dynamics or adaptive internal state, which are the core properties that LNNs are designed to provide. This contrast allows the comparison to directly isolate and demonstrate the benefit of continuous-time temporal adaptation, without introducing confounding factors. To strengthen the baseline comparison of the proposed model, three additional baseline models were evaluated including LSTM, GRU, and CNN-LSTM. LSTM and GRU were selected because they are the most commonly used recurrent architectures for DDoS detection in existing literature and possess their own form of temporal processing, allowing a meaningful comparison against models that also handle sequential data. CNN-LSTM was included as a representative hybrid architecture that combines spatial feature extraction with temporal modeling and reflects the current state of practice in IDS.

Inclusion of multiple latest datasets enables for validation and testing across multiple datasets, which is critical for measuring generalization performance. As evolution in DDoS attacks is increasing gradually so, latest datasets are more appropriate for this study. Table 2 presents main statistics for three datasets, including number of samples, number of features, and class distribution. To perform fair evaluation across all three datasets, same cleaning, preprocessing and feature selection steps were performed. Duplicate records, missing values, and highly correlated features were dropped. Each dataset was split into training and testing sets, 70% for training and 30% for testing. Feature standardization was performed using StandardScaler to normalize all input values to a zero mean and unit variance. This step prevents features with larger numeric ranges from dominating learning process.

Following preprocessing, each dataset was organized into temporal sequences to enable the LTC layers to model time-varying traffic dynamics. Since each dataset provides pre-extracted flow-level features rather than raw packets, individual flow records were ordered chronologically by their timestamp field and grouped into fixed-length sliding windows of 10 consecutive flow records, with a stride of 1, producing overlapping sequences that preserve the temporal continuity of network traffic. Each sequence therefore has the shape (10, n), where 10 is the number of time steps and n is the number of features in the respective dataset (22 for SNT-SDN2025, 40 for CICIoT2023, and 23 for IoT23). The label assigned to each sequence corresponds to the label of its final flow record, following the standard convention for sequential classification tasks. This windowing approach ensures that the LTC layers receive structured temporal context at each forward pass, allowing them to capture short-term fluctuations as well as gradual shifts in traffic behavior that are characteristic of evolving DDoS attacks. No cross-dataset feature alignment was performed, as each dataset was trained and evaluated independently to preserve the integrity of its original feature space.

Because overlapping sliding windows (window size = 10, stride = 1) were used to generate temporal sequences, special care was taken to prevent information leakage between training and testing data. For each dataset, traffic records were first sorted chronologically according to their timestamp field and then divided into non-overlapping training (70%) and testing (30%) partitions using their temporal order. Sliding-window sequence generation was subsequently performed independently within each partition. So, no sequence in the training set shared any flow records with sequences in the testing set, eliminating overlap between partitions and preventing temporal leakage. This ensures that the model is always evaluated on future unseen traffic patterns and provides a more realistic assessment of generalization performance in practical deployment scenarios.

**Table 2:** Dataset characteristics.

Dataset	Environment	Shape	Normal traffic (%)	DDoS attacks (%)
SNT-SDN2025^23^	SDN	(1034668, 22)	50.99	49.01
CICIoT2023^22^	IoT	(1555459, 40)	57.08	42.92
IoT23^24^	IoT	(1327318, 23)	51.9	48.1

### Proposed model design and training

The proposed model is based on LNN architecture to detect DDoS attack in IoT and SDN environments. Proposed model captures temporal dynamics of network traffic through liquid time constant cells (LTC). These cells model continuous time recurrent dynamics, allowing network to adapt to rapidly changing input sequences that are typical in IoT and SDN traffic. Proposed model dynamically adjusts its internal time constants to deal with complex temporal and causal relationship in network traffic. Each layer of model is the built using a LTC, that updates its hidden state based on both current input and prior hidden state dynamics. For a given time step *t*, hidden state is computed as:

4 $$\:{h}_{t}=\:{e}^{\raisebox{1ex}{$-1$}\!\left/\:\!\raisebox{-1ex}{$\left|\tau\:\right|$}\right.}{h}_{t-1}+(1-{e}^{\raisebox{1ex}{$-1$}\!\left/\:\!\raisebox{-1ex}{$\left|\tau\:\right|$}\right.})tanh({W}_{x}{x}_{t}+{W}_{h}{h}_{t-1})$$

where $\:x_{t} \in \mathbb{R}^{{\mathrm{n}}}$ is the input feature vector at time step *t*, with *n* denoting the number of input features (*n* = 22 for SNT-SDN2025, *n* = 40 for CICIoT2023, and *n* = 23 for IoT23); $\:{h}_{t}$, $\:h_{{t - 1}} \in \mathbb{R}^{{\mathrm{d}}}$ are the hidden state vectors at time steps *t* and *t − 1* respectively, with d = 32 denoting the hidden dimension applied uniformly across all three LTC layers; $\:W_{x} \in \mathbb{R}^{{{\mathrm{d}} \times {\mathrm{n}}}}$ is the input weight matrix projecting the n-dimensional input into the d-dimensional hidden space; $\:W_{h} \in \mathbb{R}^{{{\mathrm{d}} \times {\mathrm{d}}}}$ is the hidden-to-hidden weight matrix operating within the d-dimensional hidden space$\:;\:\tau \in \mathbb{R}^{{\mathrm{d}}}$is a learnable time-constant vector with one adaptive time constant per hidden unit; and tanh: $\:\mathbb{R}^{{\mathrm{d}}} \to \mathbb{R}^{{\mathrm{d}}}$ is the element-wise hyperbolic tangent activation function. For the proposed model, the input sequence per sample has shape (B × 10 × n), where B is the batch size and 10 is the fixed sliding window length, and the hidden state sequence output by each LTC layer has shape (B × 10 × 32).

To ensure clarity, each component of the proposed model is described individually in the order in which it processes the input. Network traffic features are first standardized through a batch normalization layer to ensure stable training across the heterogeneous feature distributions of the three datasets. The normalized input is then passed sequentially through three stacked LTC layers, where each layer updates its hidden state according to the continuous-time dynamics defined in Eq. 4. The hidden state sequence produced by the final LTC layer is forwarded to the multi-head self-attention module, which reweights temporal dependencies across the sequence as defined in Eq. 5. The attended output is passed through layer normalization and a dropout layer before entering the fully connected classification layers, which produce final class probabilities via a log-softmax activation.

The overall architecture consists of:

- Input normalization using batch normalization.
- Stacked LTC layers: Three layers with a hidden dimension of 32 neurons each.
- Multi-head self-attention with four heads to enhance temporal feature representation and highlight relevant traffic patterns.
- Fully connected output layers for classification, followed by a log-softmax activation to produce probabilistic outputs across two classes (benign vs. DDoS).
- Layer normalization and dropout applied before final prediction layer to stabilize training and improve generalization.

The attention mechanism is defined in Eq. (5): This attention mechanism is a structural component of the proposed LNN model and is not related to the adversarial evaluation methodology. It operates during both training and inference to improve the model’s ability to focus on critical temporal dependencies within network traffic flows.

5 $$\:MultiHead\left(Q,K,V\right)=Concat({head}_{1},\dots\:,{head}_{h}){W}^{o}$$

where $$\:{head}_{i}=Softmax\left(\frac{\left(Q{W}_{i}^{o}\right)(K{W}_{i}^{K}{)}^{T}}{\left(\sqrt{{d}_{k})}\:\right(V{W}_{i}^{v})}\right)$$.

where h = 4 is the number of attention heads, $\:Q,\:K,\:V\in\:\:{\mathbb{R}}^{B\times\:10\times\:32}\:$are the query, key, and value matrices derived from the LTC layer output; for each head *i*, $\:{W}_{i}^{o}$, $\:{W}_{i}^{K}$, $\:{W}_{i}^{v}\in\:{\mathbb{R}}^{32\times\:8}$, are learned per-head projection matrices that independently project the shared input into a lower-dimensional subspace of dimension $\:{d}_{k}={d}_{v}=\frac{d}{h}=\frac{32}{4}$ = 8, scaled dot-product attention is computed independently for each head using these projections; the four resulting head outputs, each of shape (B × 10 × 8), are concatenated to form a tensor of shape (B × 10 × 32) and projected by $\:{W}^{o}\in\:{\mathbb{R}}^{32\times\:32}$ to produce the final attended output of shape (B × 10 × 32), and division by $\:\sqrt{{d}_{k}}$ prevents dot products from growing too large in high-dimensional spaces, which would push the softmax into regions of extremely small gradients. Attended features are normalized using a layer normalization operation and passed through fully connected layers. This allows model to focus on most relevant temporal dependencies across sequence. The final output is produced by a log-softmax activation that estimates class probabilities for binary classification.

The integration of multi-head attention after the stacked LTC layers provides two specific benefits in the context of DDoS detection. First, it enables the model to selectively weight the most discriminative temporal positions within a traffic flow sequence, which is critical when attack patterns are embedded within otherwise normal traffic. Second, by allowing each of the four attention heads to independently focus on different temporal sub-spaces, the mechanism captures diverse co-occurrence patterns among traffic features, such as the simultaneous shift in packet rate and flow duration that characterizes DDoS flooding, that a single attention head would fail to isolate. The contribution of this mechanism is empirically confirmed in the ablation study, where its removal causes the largest single accuracy drop of approximately 5.02% across all three datasets.

Negative log-likelihood loss (NLLLoss) function is given in Eq. (6):

6 $$\:L=\:-\frac{1}{N}\:\sum\:_{i=1}^{N}\sum\:_{c=1}^{C}{y}_{i,c}log\left({Y}_{i,c}\right)$$

Where * N* is the total number of training samples, * C* is the number of output classes (C = 2 for binary DDoS classification), * i* is the sample index, * c* is the class index, $\:{y}_{i},c$ is the ground truth label for sample * i* and class * c*, and $\:{Y}_{i},c$ is the predicted log-softmax probability for sample * i* belonging to class * c*.

Adam (Adaptive Moment Estimation) optimizer was used to minimize NLLLoss. Adam combines benefits of Adaptive Gradient (AdaGrad) and Root Mean Square Propagation (RMSProp) by maintaining running estimates of both first and second moments of gradients. Where $\:{m}_{t}$ is the first moment estimate (exponential moving average of gradients) at iteration * t*, $\:{v}_{t}$ is the second moment estimate (exponential moving average of squared gradients) at iteration * t*, $\:{M}_{t}$ and $\:{V}_{t}$ are the bias-corrected first and second moment estimates respectively, * t* is the current iteration number used for bias correction, $\:{g}_{t}$ is the gradient of the loss with respect to parameter $\:{\theta\:}_{t}$, $\:\alpha\:$ is the learning rate, $\:{\beta\:}_{1}$ and $\:{\beta\:}_{2}$ are exponential decay rates for the first and second moment estimates, and ε is a small constant added for numerical stability. The parameter update at iteration *t* is defined in Eqs. (7), (8), (9), (10), and (11):

7 $$\:{m}_{t}={\beta\:}_{1}{m}_{t-1}+(1-{\beta\:}_{1}){g}_{t}$$

8 $$\:{v}_{t}={\beta\:}_{2}{v}_{t-1}+(1-{\beta\:}_{2}){g}_{t}^{2}$$

9 $$\:{M}_{t}=\frac{{m}_{t}}{1-{\beta\:}_{1}^{t}}$$

10 $$\:{V}_{t}=\frac{{v}_{t}}{1-{\beta\:}_{2}^{t}}$$

11 $$\:{\theta\:}_{t+1}={\theta\:}_{t}-\alpha\:\frac{{M}_{t}}{\sqrt{{V}_{t}+\epsilon\:}}$$

Where $\:{g}_{t}$ is gradient of loss with respect to parameter $\:{\theta\:}_{t}$, α is learning rate, $\:{\beta\:}_{1}$and $\:{\beta\:}_{2}$ are exponential decay rates for the first and second moment estimates, and ε is a small constant for numerical stability. In this research work, learning rate $\:\alpha\:$ was set to 0.001, with default values $\:{\beta\:}_{1}=0.9$, $\:{\beta\:}_{2}=0.999$, and $\:\varepsilon = 10^{{ - 8}}$.

It is important to distinguish between the two types of attacks addressed in this study, as they differ fundamentally in their nature and purpose. DDoS attacks are real network-level threats in which malicious actors overwhelm IoT devices or SDN controllers by flooding them with high volumes of traffic, varying packet rates, flow durations, and attack sources to evade detection. These are the primary threat that the proposed model is trained to detect and classify. Adversarial attacks, on the other hand, are not network attacks in the traditional sense. They are deliberately crafted input perturbations applied to the feature vectors of traffic samples to deceive a trained machine learning model into misclassifying malicious traffic as benign. In this study, adversarial attacks are generated using the MIM framework exclusively as a post-training robustness evaluation tool, they simulate a sophisticated attacker who not only generates DDoS traffic but also knows how to subtly modify its statistical features to bypass the detection model. The proposed model must therefore be robust to both, it must accurately classify DDoS traffic under normal conditions, and it must resist feature-level perturbations designed to fool it under adversarial conditions.

It is important to note that the Momentum Iterative Method (MIM) is an adversarial attack technique used exclusively for robustness evaluation, and is entirely distinct from the multi-head attention mechanism, which is an internal architectural component of the proposed model used for feature representation during training and inference. These two techniques serve fundamentally different purposes and operate at different stages of the experimental pipeline. To assess the robustness and generalization of proposed model, MIMO adversarial attack based on the MIM was applied. The goal was to generate small but targeted perturbations to input samples in order to test model’s ability to resist evasion attempts. The MIM attack extends fast gradient sign method (FGSM) by incorporating iterative updates and a momentum term to stabilize gradient directions. It is stronger than FGSM attacks.

MIM was selected over other adversarial attack methods for three specific reasons. First, MIM is a gradient-based iterative attack that is significantly stronger than single-step methods such as FGSM, making it a more rigorous test of model robustness. Second, MIM’s momentum term stabilizes gradient directions across iterations, which makes the generated perturbations more transferable across different model architectures, a property that is particularly relevant when comparing robustness across different models as done in this study. Third, MIM has been widely used in adversarial robustness evaluation of deep learning models in security-critical applications, making it a well-established and reproducible benchmark for comparing adversarial resilience. Compared to more complex attacks such as the Carlini and Wagner (C&W) attack or AutoAttack, MIM offers a practical balance between attack strength and computational feasibility, which is important given the large scale of the three datasets used in this study. Given an input sample *X*, label *y*, and model parameters $\:\theta\:$, adversarial example $\:{X}^{adv}$ is generated iteratively as given in Eqs. (12) and (13):

12 $$\:{g}_{t+1}=\mu\:{g}_{t}+\frac{\nabla\:{x}^{J}(\theta\:,{X}_{t}^{adv},y)}{\left|\right|{\nabla\:{x}^{J}(\theta\:,X}_{t}^{adv},y\left)\right|{|}_{1}}$$

13 $$ \:X_{{t + 1}}^{{adv}} = Clip_{{x,\varepsilon }} (X_{t}^{{adv}} + \alpha \cdot sign(g_{{t + 1}} )) $$

Where:

- $\:{g}_{t}$ is accumulated gradient at iteration *t* with momentum coefficient μ = 0.9.
- α represents step size for each iteration.
- ε bounds maximum perturbation allowed per sample.
- $\:Clip_{{x,\varepsilon }}$ (Z) is the element-wise clipping operator defined as: $\:{Clip}_{x,\varepsilon}\:\left(Z\right)=\mathrm{m}\mathrm{i}\mathrm{n}(X+\varepsilon,\:\mathrm{m}\mathrm{a}\mathrm{x}(X-\varepsilon,\:Z\left)\right)$ where $\:X\in\:{\mathbb{R}}^{n}$ is the original clean input sample, $\:Z\in\:{\mathbb{R}}^{n}$ is the perturbed candidate, and ε = 0.1 is the maximum allowed perturbation per feature. The operator constrains each feature of the adversarial example to remain within the interval [X − ε, X + ε], ensuring that the perturbation is bounded and that the perturbed sample remains within a plausible ε-neighborhood of the original input. Applied element-wise across all *n* features, it guarantees that $\:{X}^{{\left(t+1\right)}_{\_adv}}$ satisfies $\:{\Vert\:X}^{{\left(t+1\right)\_adv}_{\:}}-X\Vert\:\infty\:\le\:\varepsilon$ at every iteration.

To avoid convergence to local minima, attack is executed with multiple random restarts, each adding small uniform noise within the range [−ε, ε]. The hyperparameters used for attack were:

- Perturbation limit (ε = 0.1): This value bounds the maximum perturbation allowed per feature. A value of 0.1 was chosen because it is large enough to produce meaningful perturbations that challenge the model, while remaining small enough to preserve the statistical plausibility of the traffic samples. Values significantly larger than 0.1 would produce unrealistic feature distributions that would not represent genuine evasion attempts in real network traffic.
- Step size (α = 0.002): The step size was set to one-fiftieth of the perturbation limit, following the standard convention in iterative adversarial attack literature. This ratio ensures that the attack makes meaningful progress per iteration without overshooting the ε-bounded perturbation space.
- Iterations = 10: Ten iterations were found to be sufficient for the attack to converge within the ε-bounded perturbation space across all three datasets. Increasing iterations beyond 10 produced negligible additional accuracy drop in preliminary experiments, confirming that 10 iterations represent a practical and effective setting.
- Restarts = 3: Multiple random restarts were used to avoid convergence to local minima. Three restarts provided a good balance between attack thoroughness and computational cost. Each restart initializes from a different random perturbation within the ε-neighborhood, increasing the probability of finding the most effective adversarial example.

The adversarial accuracy was measured by evaluating trained model on adversarially perturbed validation data. The metric is given in Eq. (14):

14 $$\:Adversarial\:Accuracy=\:\frac{1}{N}={\sum\:}_{i=1}^{N}1({Y}_{i}^{adv}={y}_{i})$$

Where * N* is the total number of evaluation samples, $\:{Y}_{i}^{adv}$ is the model’s predicted label for adversarially perturbed input $\:{X}_{i}^{adv}$, $\:{y}_{i}$ is the true ground truth label for sample * i*, and $\:1(\cdot)$ is the indicator function that equals 1 when the predicted label matches the true label and 0 otherwise.

**Fig. 1:**
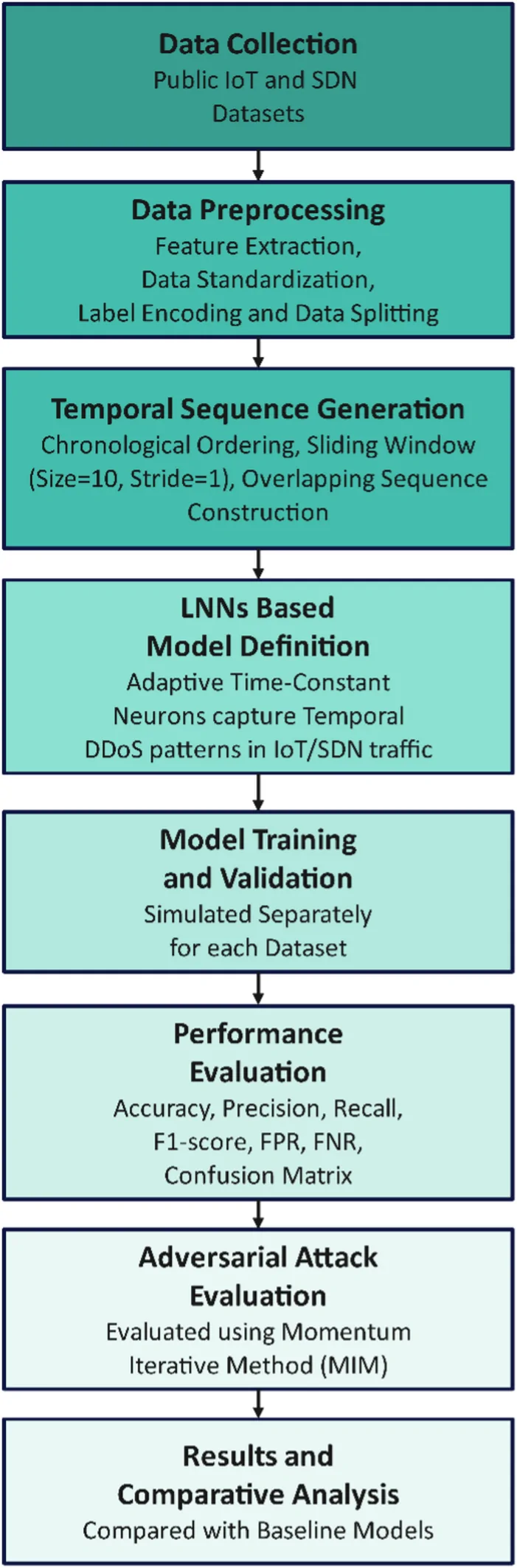
Workflow of the proposed LNNs based DDoS detection framework.

**Fig. 2:**
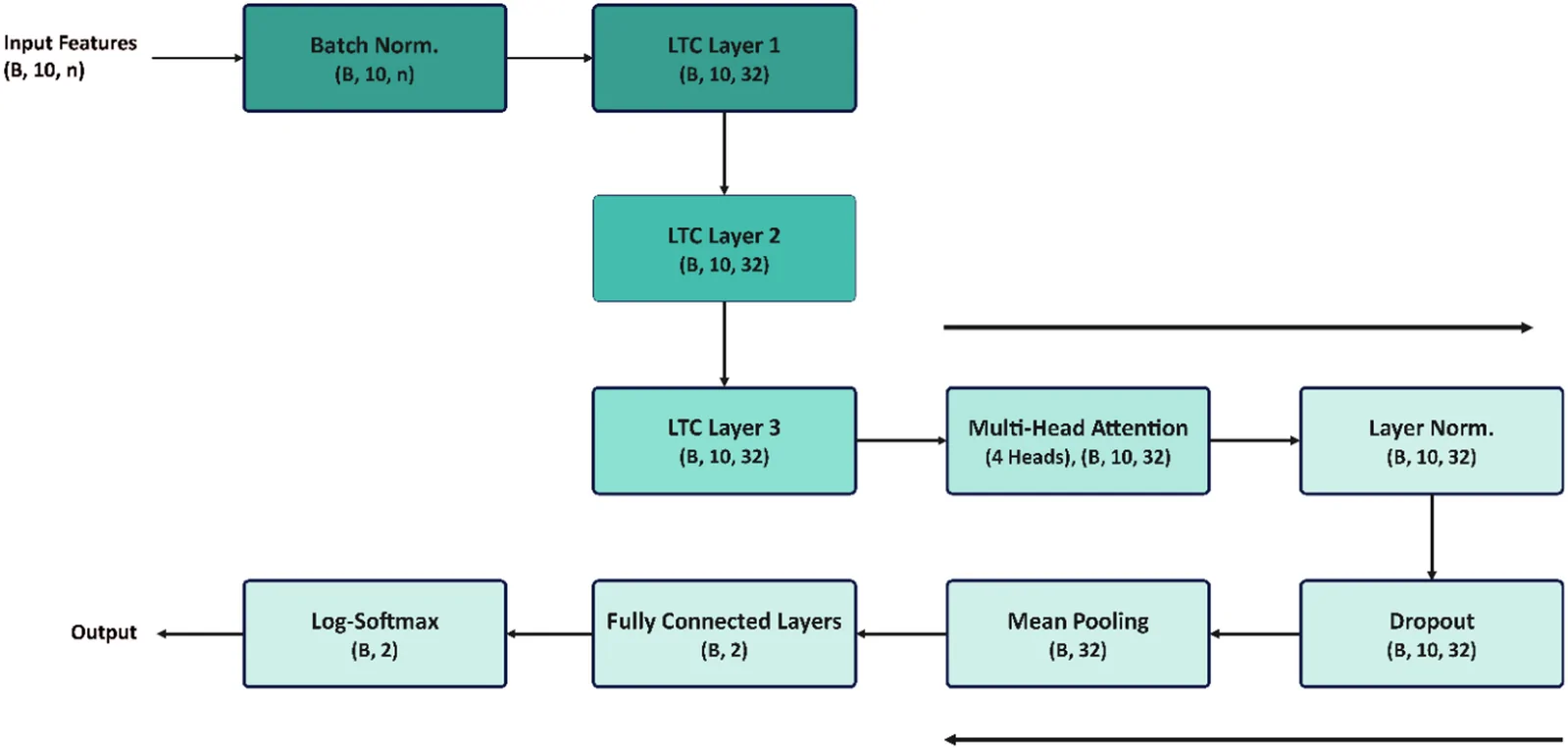
Proposed model architecture.

This simulation setup produced challenging adversarial attacks that effectively tested model’s defense against adaptive perturbations in IoT and SDN traffic patterns. Figure 1 shows basic work flow of proposed model and Fig. 2 shows system architecture of proposed model. Figure 2 illustrates the internal architecture of the proposed model and intentionally does not include the MIM-based adversarial evaluation pipeline. This is because MIM is an external robustness testing procedure applied independently after training is complete, and is not a component of the model architecture itself.

### Hyperparameters

Proposed model’s hyperparameters were selected analytically after performing multiple training rounds to balance detection accuracy, convergence stability, and computational efficiency. Combination of a moderate hidden size and three LTC layers allowed proposed model to deal with nonlinear temporal dependencies in IoT and SDN traffic. Proposed model uses multiple regularization and attention mechanisms to improve generalization and the representation learning. The Dropout rate of 0.2 was applied within each LTC to prevent co-adaptation among recurrent units. Higher dropout rate of the 0.4 was used in fully connected layers following attention segment. Multi-head attention mechanism uses four attention heads, allowing model to catch multiple temporal and feature level dependencies at once. Adam optimizer help to stabilize training in neural networks with complex temporal dynamics for example, LNNs. Learning rate was set at 0.001 after experimental testing with Adam optimizer. The proposed model trained using a batch size of 256 which provided an appropriate balance between computational efficiency and stable gradient estimation. This allowed model to converge effectively without exceeding memory limits. Training was conducted using a maximum of 30 epochs which allowed model sufficient iterations to learn statistical patterns in traffic data. To avoid overfitting, an early stopping mechanism with a patience of seven epochs was used. Table 3 summarizes final configuration used for all experiments. The hyperparameter configurations were determined through multiple rounds of experimentation and applied uniformly across all three datasets, rather than being independently tuned per dataset. This was done to evaluate the generalizability of the proposed architecture under a fixed configuration rather than dataset-specific optimization.

**Table 3:** Hyperparameter configuration.

Parameter	Description	Value
Hidden Size	Number of neurons in each LTC layer	32
Number of Layers	Total stacked LTC layers	3
Dropout (LTC)	Dropout rate within each LTC cell	0.2
Dropout (Fully connected)	Dropout applied to the final dense layers	0.4
Attention Head	Number of heads in the multi-head attention module	4
Learning Rate	Step size for the Adam optimizer	0.001
Optimizer	Optimization algorithm used during training	Adam
Batch Size	Number of samples per training batch	256
Epochs	Maximum number of training epochs	30
Early Stopping Patience	Epochs without improvement before stopping	7

## Simulation results

**Fig. 3:**
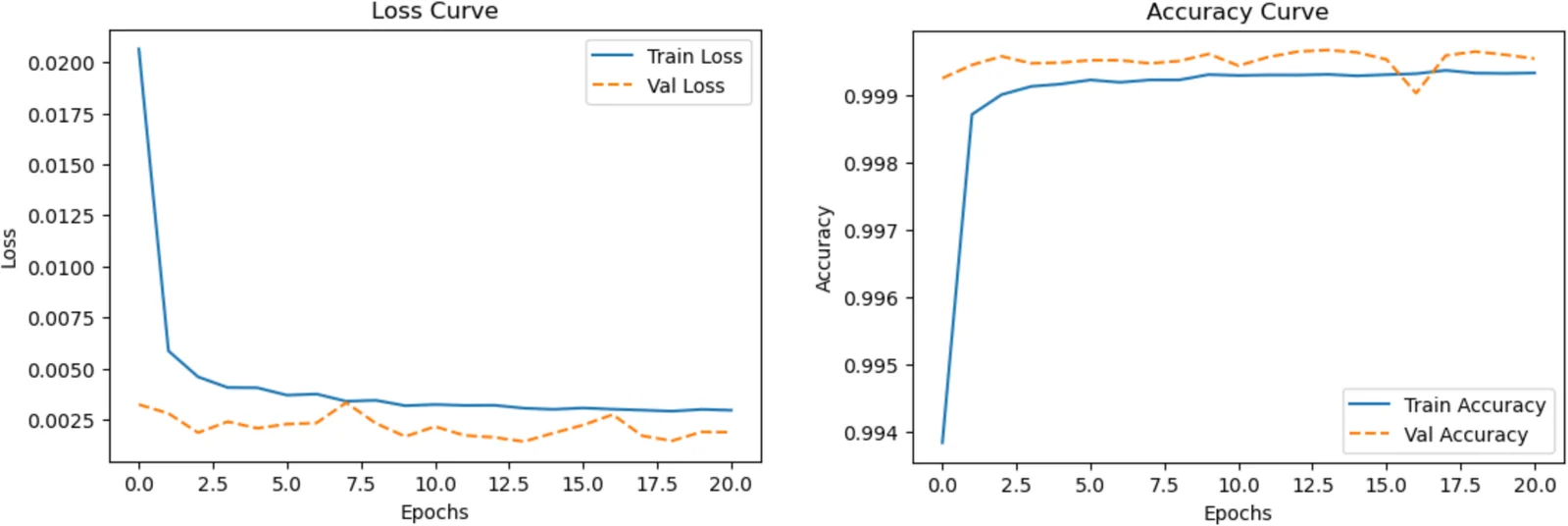
Training loss, validation loss, training accuracy and validation accuracy using SNT-SDN2025.

**Fig. 4:**
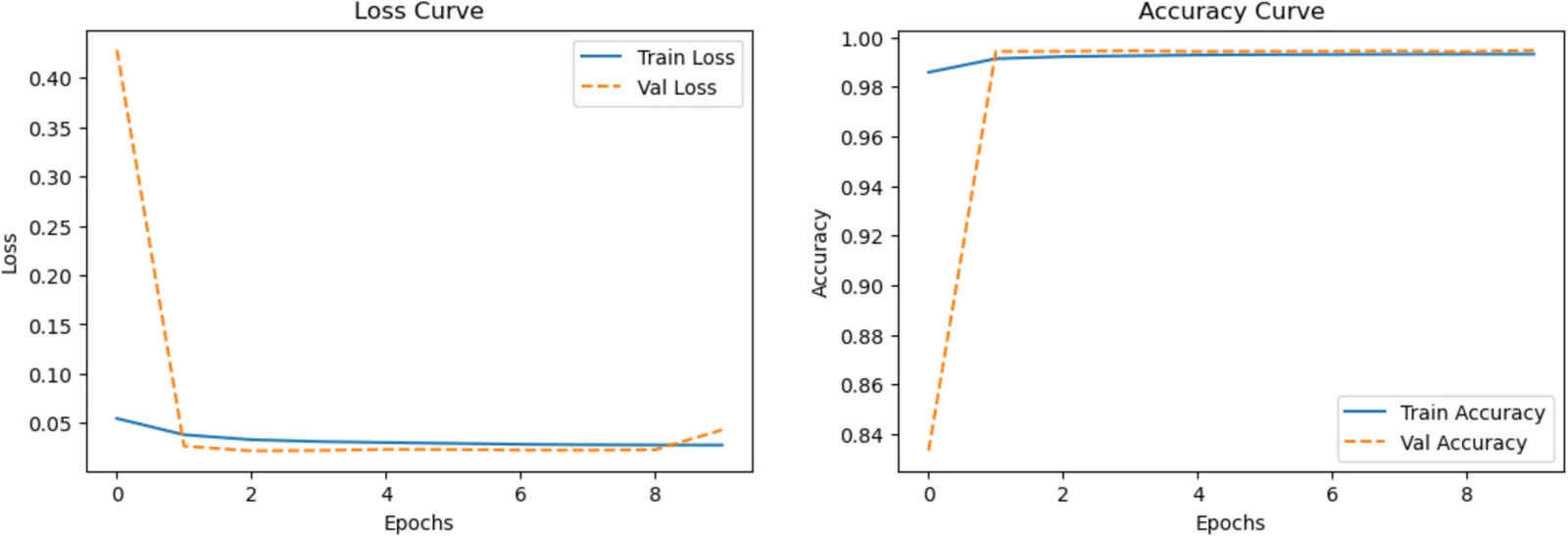
Training loss, validation loss, training accuracy and validation accuracy using CICIoT2023.

**Fig. 5:**
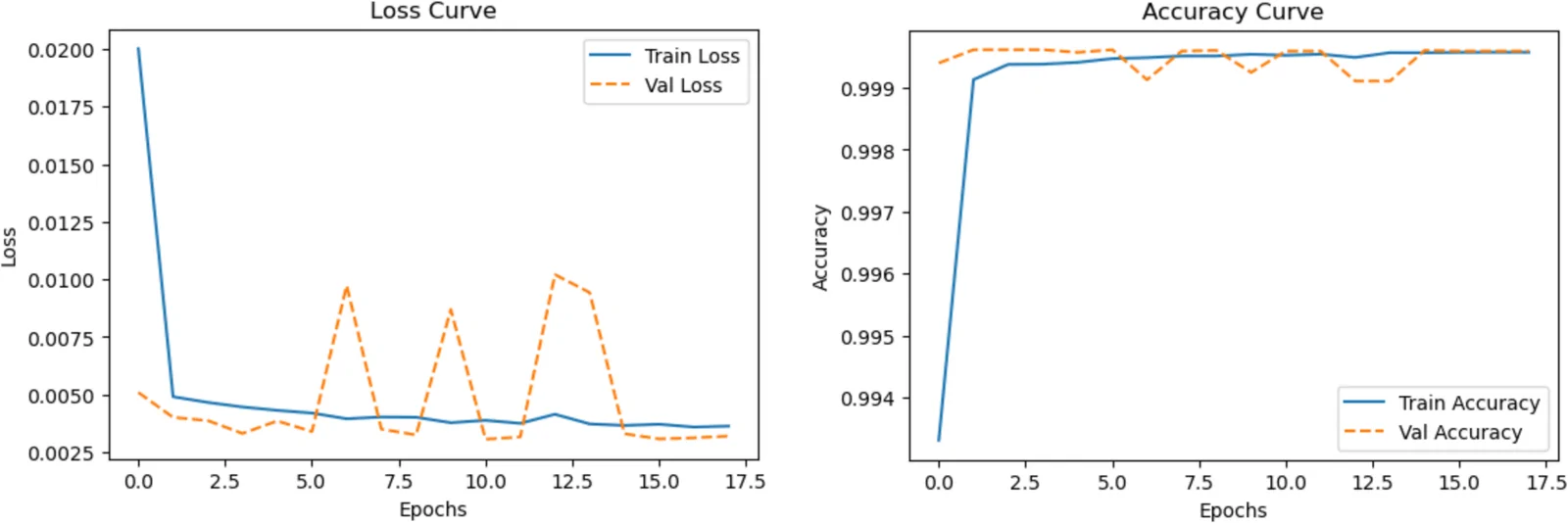
Training loss, validation loss, training accuracy and validation accuracy using IoT23.

**Fig. 6:**
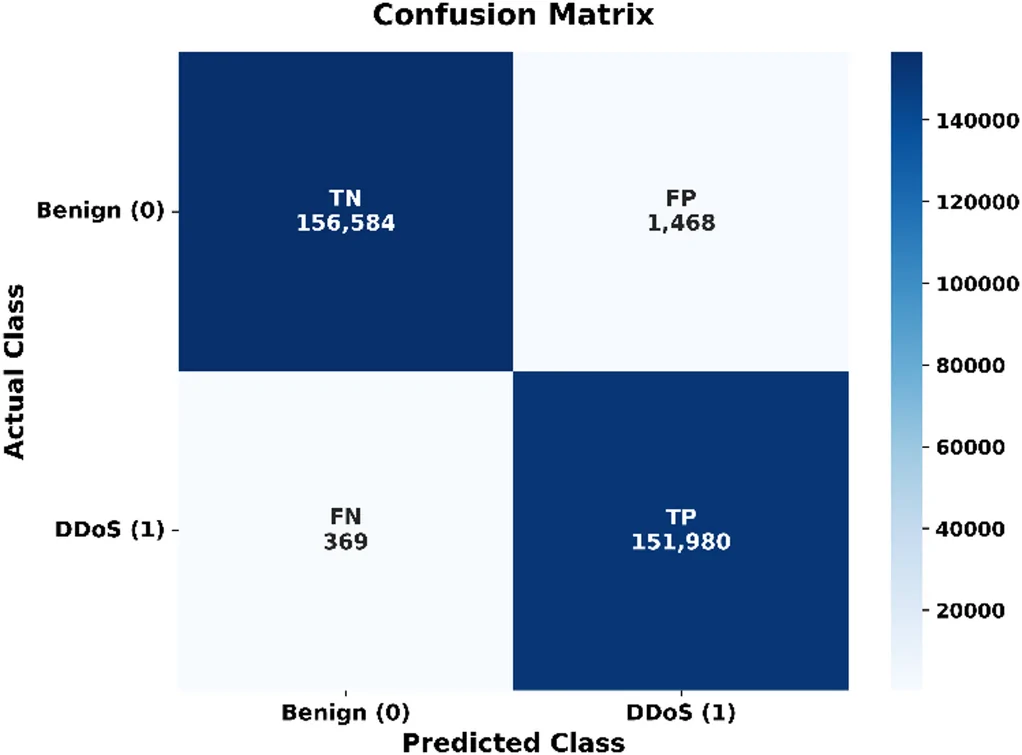
Confusion matrix using SNT-SDN2025.

**Fig. 7:**
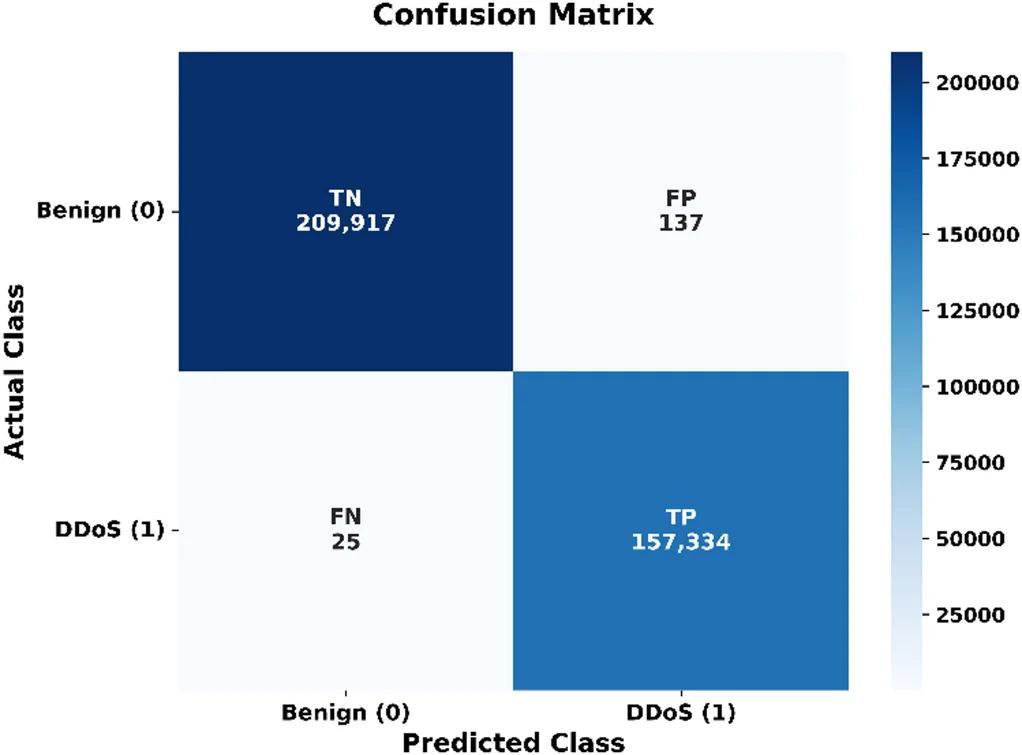
Confusion matrix using CICIoT2023.

**Fig. 8:**
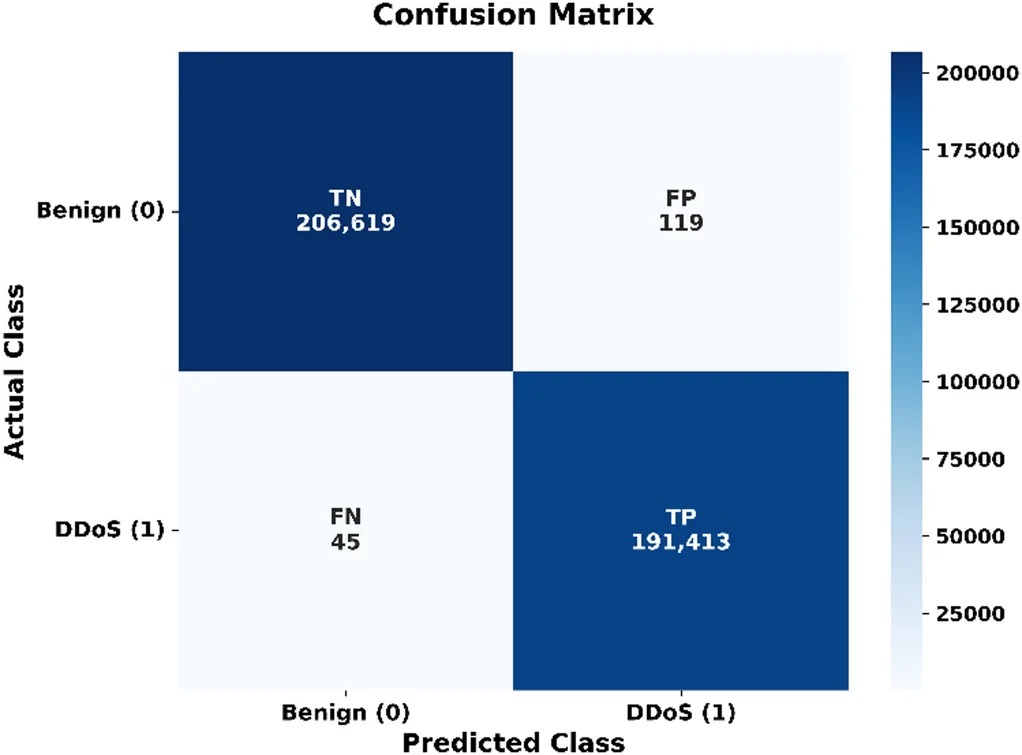
Confusion matrix using IoT23.

The proposed model has been simulated using Python, Pandas, NumPy, Matplotlib, seaborn, PyTorch, and scikit-learn (SKLearn). The system used for simulations has specification of 8GB RAM, Intel Core i5-8300U CPU @ 1.70 GHz, and 256GB solid-state drive (SSD). To validate performance of proposed model, evaluation metrics including accuracy score, precision, recall, and F1-score are used. The proposed model was tested and evaluated using three datasets. The proposed model gave highest accuracy score (99.96%) on CICIoT2023 dataset with FPR of 0.07% and FNR rate of 0.02%. The same simulation environment was used to evaluate all baseline models including MLP, LSTM, GRU, and CNN-LSTM. Among the baselines, CNN-LSTM achieved the highest accuracy of 97.84% on IoT23, while MLP achieved the lowest at 93.30% on CICIoT2023. Table 4 summarizes the score of evaluation metrics for all models. All models were trained and evaluated across five independent runs using different random seeds. Table 4 reports the mean and standard deviation of each metric across these runs to confirm the statistical reliability of the reported results. To support the Table 4, loss and accuracy curves are included. Figures 3, 4 and 5 show the training loss, validation loss, training accuracy and validation accuracy for proposed model on SNT-SDN2025, CICIoT2023 and IoT23 datasets respectively. To further support the claim for the performance of proposed model, confusion matrices are also included. Confusion matrix is used to visualize the performance of a classification model, showing how many samples were correctly and incorrectly classified. Figures 6, 7 and 8 show the confusion matrices for proposed model on SNT-SDN2025, CICIoT2023 and IoT23 datasets respectively.

**Table 4:** Performance comparison of the proposed and baseline models.

Dataset	Model	Accuracy	Precision	Recall	F1-Score	FPR	FNR
SNT-SDN2025	Proposed Model	99.43% ± 0.03%	99.41% ± 0.04%	99.45% ± 0.03%	99.44% ± 0.03%	0.71% ± 0.02%	0.24% ± 0.01%
CICIoT2023	Proposed Model	99.96% ± 0.02%	99.94% ± 0.02%	99.96% ± 0.02%	99.95% ± 0.02%	0.07% ± 0.01%	0.02% ± 0.01%
IoT23	Proposed Model	99.75% ± 0.04%	99.71% ± 0.05%	99.72% ± 0.04%	99.73% ± 0.04%	0.14% ± 0.02%	0.10% ± 0.01%
SNT-SDN2025	MLP	93.65% ± 0.12%	93.67% ± 0.13%	93.61% ± 0.11%	93.63% ± 0.12%	3.4% ± 0.08%	2.5% ± 0.07%
CICIoT2023	MLP	93.30% ± 0.14%	93.25% ± 0.15%	93.21% ± 0.13%	93.24% ± 0.14%	3.6% ± 0.09%	2.9% ± 0.08%
IoT23	MLP	93.37% ± 0.13%	93.36% ± 0.14%	93.34% ± 0.12%	93.30% ± 0.13%	3.5% ± 0.09%	3.1% ± 0.08%
SNT-SDN2025	LSTM	93.74% ± 0.11%	93.73% ± 0.12%	93.61% ± 0.10%	93.64% ± 0.11%	3.3% ± 0.08%	3.1% ± 0.07%
CICIoT2023	LSTM	93.90% ± 0.12%	93.97% ± 0.13%	93.82% ± 0.11%	93.80% ± 0.12%	3.1% ± 0.07%	2.9% ± 0.07%
IoT23	LSTM	93.85% ± 0.13%	93.81% ± 0.14%	93.84% ± 0.12%	93.87% ± 0.13%	2.9% ± 0.07%	3.0% ± 0.07%
SNT-SDN2025	GRU	95.37% ± 0.09%	95.15% ± 0.10%	95.16% ± 0.09%	95.10% ± 0.09%	2.8% ± 0.07%	2.2% ± 0.06%
CICIoT2023	GRU	94.67% ± 0.11%	94.14% ± 0.12%	94.07% ± 0.10%	94.01% ± 0.11%	3.2% ± 0.08%	2.1% ± 0.06%
IoT23	GRU	95.95% ± 0.08%	95.75% ± 0.09%	95.55% ± 0.08%	95.41% ± 0.09%	2.8% ± 0.06%	2.3% ± 0.05%
SNT-SDN2025	CNN-LSTM	97.16% ± 0.07%	96.93% ± 0.08%	96.63% ± 0.07%	96.51% ± 0.07%	2.5% ± 0.06%	1.9% ± 0.05%
CICIoT2023	CNN-LSTM	97.25% ± 0.08%	97.09% ± 0.09%	97.10% ± 0.07%	96.97% ± 0.08%	2.1% ± 0.05%	1.4% ± 0.04%
IoT23	CNN-LSTM	97.84% ± 0.06%	97.77% ± 0.07%	97.71% ± 0.06%	97.67% ± 0.06%	2.3% ± 0.05%	1.6% ± 0.04%

To evaluate the effect of MIM-based adversarial perturbations on model performance, all models were tested on adversarially perturbed versions of the validation data. The MIM attack generated subtle but targeted perturbations within a bounded epsilon neighborhood of the original input samples, simulating realistic evasion attempts by adversarial attackers. Under these conditions, the proposed LNN model experienced accuracy drops of 8.20%, 7.81%, and 8.82% on SNT-SDN2025, CICIoT2023, and IoT23 datasets respectively, while retaining adversarial accuracy above 90% across all three datasets. In contrast, MLP suffered drops of 19.34%, 21.41%, and 20.71%, LSTM dropped by 15.29% to 16.00%, GRU by 14.26% to 15.18%, and CNN-LSTM by 12.46% to 12.89%. These results demonstrate that MIM perturbations had a substantially smaller impact on the proposed model compared to all baselines, confirming that the continuous-time temporal dynamics of LNNs provide inherent resistance to gradient-based adversarial manipulation.

To evaluate the individual contribution of each architectural component of the proposed model, an ablation study was conducted. Four model variants were derived from the full proposed model by removing one component at a time: the multi-head attention mechanism, two of the three stacked LTC layers (reducing to a single layer), the input batch normalization, and the dropout regularization. Each variant was trained and evaluated under identical conditions across all three datasets. Table 5 summarizes the accuracy and F1-score results of the ablation study.

**Table 5:** Ablation study of proposed model components.

Model Variant	SNT-SDN2025 (Accuracy)	SNT-SDN2025 (F1-Score)	CICIoT2023 (Accuracy)	CICIoT2023 (F1-Score)	IoT23 (Accuracy)	IoT23 (F1-Score)
Full Model	99.43%	99.44%	99.96%	99.95%	99.75%	99.73%
Without Attention	94.74%	94.63%	94.97%	94.90%	94.35%	94.25%
Single LTC Layer	96.88%	96.71%	96.91%	96.87%	96.45%	96.35%
Without Batch Norm	97.21%	97.16%	97.56%	97.45%	97.33%	97.30%
Without Dropout	98.53%	98.51%	98.77%	98.75%	98.44%	98.39%

The ablation results confirm that each component of the proposed model contributes meaningfully to its overall performance. Removing the multi-head attention mechanism resulted in the largest accuracy drop, reducing average accuracy from 99.71% to 94.69%, a drop of approximately 5.02% across all three datasets, demonstrating that temporal feature representation is critical for capturing the complex dependencies in DDoS traffic. Reducing the architecture to a single LTC layer caused an average accuracy reduction of approximately 2.79%, with accuracy falling to 96.75% on average, confirming that depth in LTC stacking is necessary to model the nonlinear temporal dynamics of IoT and SDN traffic. Removing batch normalization led to an average accuracy drop of approximately 2.35%, reducing average accuracy to 97.37%, indicating that input normalization is important for stable training across the heterogeneous feature distributions of the three datasets. Disabling dropout resulted in the smallest average drop of approximately 1.12%, with average accuracy reaching 98.58%, suggesting that regularization contributes to generalization although early stopping partially compensates for its absence. These results validate the design choices made in the proposed model and demonstrate that the full architecture achieves the best balance between accuracy, generalization, and robustness.

## Discussion

The MLP baseline was deliberately chosen as a representative static architecture to highlight the performance gap introduced by the absence of temporal adaptation, which is the fundamental limitation that the proposed LNN-based model addresses. Furthermore, other baseline models including LSTM and GRU incorporate recurrent temporal processing, they rely on discrete time steps and fixed hidden state update rules, which limits their ability to adapt to the continuous and irregular temporal dynamics characteristic of DDoS traffic in IoT and SDN environments. The proposed LNN model outperformed all baselines including CNN-LSTM, which represents the strongest conventional hybrid architecture evaluated, demonstrating that continuous-time adaptive computation provides a meaningful advantage beyond what standard recurrent models can achieve. The experimental results of this study show that proposed model give significant improvement as compared to baseline models. Unlike the DDoS attacks evaluated in Table 4, which represent real network-level flooding threats, the adversarial attacks evaluated in Table 6 represent feature-level perturbations designed to deceive the detection model itself, a fundamentally different and complementary threat scenario. Table 6 shows that the proposed model provides adversarial accuracy above 90% on all datasets whereas baseline model fails to provide good performance and its accuracy drops around 72% when adversarial attacks applied to it. When adversarial attacks applied to proposed model, the accuracy drop was only around 9% whereas in case of baseline model it was around 20%.

CNN-LSTM demonstrated the strongest adversarial resilience among the baselines, with accuracy drops ranging from 12.46% to 12.89% across datasets, followed by GRU at 14.26% to 15.18% and LSTM at 15.29% to 16.00%. CNN-LSTM’s adversarial accuracy reached only 84.95% at best, compared to a minimum of 90.93% for the proposed model. Notably, the proposed model is the only model that maintained adversarial accuracy above 90% across all three datasets. The marginal improvement of LSTM over MLP on the SNT-SDN2025 dataset indicates that standard recurrent architectures do not sufficiently capture the temporal complexity of SDN traffic, further motivating the use of continuous-time models. GRU’s relatively lower performance on CICIoT2023 compared to IoT23 may be attributed to the higher feature dimensionality of CICIoT2023, where GRU’s simpler gating structure is less effective at capturing complex inter-feature temporal dependencies. The MIM attack operates by accumulating momentum across iterative gradient updates, generating perturbations that are more transferable and stable than single-step attacks such as FGSM.

Despite this stronger attack formulation, the proposed model’s adaptive internal state, which continuously adjusts its temporal dynamics in response to incoming input provides a natural defense against such perturbations. Because LNN neurons update their hidden states based on continuous-time differential equations rather than fixed discrete transformations, the impact of bounded input perturbations on the final classification output is effectively attenuated, explaining the relatively small accuracy drop observed across all datasets. The improved performance is directly related to computational dynamics of LNNs. The static architectures of DL and ML do not allow them to improve their internal states with respect to time and deal with temporal dependencies in the traffic but in case of LNNs they easily handle these challenges.

The dynamic behavior of LNNs allow them adaptation to changing attack traffic of DDoS. Table 6 presents a comparative evaluation of the LNNs based proposed model and the baseline MLP model under both clean and adversarial conditions across the SNT-SDN2025, CICIoT2023, and IoT23 datasets. The proposed model demonstrates superior robustness on all datasets. The proposed model achieved highest accuracy on CICIoT2023 dataset with a lowest accuracy drop. The highest accuracy achieved by baseline model is on SNT-SDN2025 dataset with lowest accuracy drop. The minimum accuracy achieved by proposed model is on SNT-SDN2025 dataset whereas the minimum accuracy achieved by baseline model is on CICIoT2023 dataset. The highest accuracy drop by proposed model is on IoT23 dataset whereas the highest accuracy drop by baseline model is on CICIoT2023 dataset. These performance details shows that a model may have slightly different performance on different datasets. Here in the case of DDoS detection what matters the most is the minimum accuracy drop under adversarial conditions which also tells that the model have the potential to perform well even when the traffic patterns change rapidly.

**Table 6:** Comparison of model robustness under clean and adversarial conditions.

Dataset	Model	Adversarial accuracy	Clean accuracy	Accuracy drop
SNT-SDN2025	Proposed Model	91.23%	99.43%	8.20%
CICIoT2023	Proposed Model	92.15%	99.96%	7.81%
IoT23	Proposed Model	90.93%	99.75%	8.82%
SNT-SDN2025	MLP	74.31%	93.65%	19.34%
CICIoT2023	MLP	71.89%	93.30%	21.41%
IoT23	MLP	72.66%	93.37%	20.71%
SNT-SDN2025	LSTM	78.45%	93.74%	15.29%
CICIoT2023	LSTM	77.94%	93.90%	15.96%
IoT23	LSTM	77.85%	93.85%	16.00%
SNT-SDN2025	GRU	80.39%	95.37%	14.98%
CICIoT2023	GRU	80.41%	94.67%	14.26%
IoT23	GRU	80.77%	95.95%	15.18%
SNT-SDN2025	CNN-LSTM	84.56%	97.16%	12.60%
CICIoT2023	CNN-LSTM	84.79%	97.25%	12.46%
IoT23	CNN-LSTM	84.95%	97.84%	12.89%

Table 7 summarizes a comparative analysis between existing DDoS detection studies and LNN based proposed model. Table 8 further illustrates the breadth of existing LNN applications across non-security domains, reinforcing that no prior work has applied LNNs to network intrusion or DDoS detection. Most existing works have depended on traditional ML and DL algorithms such as RNN-LSTM, LSTM, SVM, DT, KNN, CNN, and GRU, achieving accuracies ranging from 89.63% to 99.84%. However, none of these studies incorporated temporal adaptation and evaluated robustness against adversarial attacks, two critical aspects in SDN and IoT environments. In contrast, LNN based proposed model demonstrates superior performance with an accuracy of 99.96%, while effectively integrating temporal adaptation to capture time-varying attack behaviors. As far as we know this is only model and framework in this comparison area which has been evaluated in presence of adversarial attacks and it proved its resilience, stability and adaptability. The discussed and mentioned results show and prove effectiveness of LNN based proposed model in accomplishing both high detection accuracy and enhanced robustness making it a promising solution for DDoS detections and mitigation in dynamic networks.

Experimental results mentioned in the Tables 4, 5 and 6 show the significance of LNNs and their potential to deal with adversarial conditions. These results also prove that why adaptive DDoS detection is necessary. From Table 4 it is worth noting that the proposed model provided lowest FPR and FNR values which also signify its potential. Low FPR and FNR shows that the model is not much effected by rapidly changing traffic and adversarial conditions. If a model is dodged by attackers by changing traffic patterns, then it can be really harmful for the system protected by model. Rapid traffic pattern change is common in the dynamic network environments which signifies the requirement and need of a detection system which can adapt to dynamic network environments rapidly. Figures 3, 4, 5, 6 and 7, and 8 also provides the evidence for the potential of proposed model against DDoS attacks. The proposed model provides almost same performance with a slight variation on all the three considered datasets. This signifies that the generalization and effectiveness of proposed model.

To assess the feasibility of deploying the proposed model in real-time IoT and SDN environments, computational complexity and inference time were measured on the CICIoT2023 dataset, which represents the largest and most feature-rich evaluation environment in this study. As reported in Table 9, the proposed model contains only 11,498 trainable parameters, the fewest among all evaluated models including MLP, demonstrating the lightweight nature of the LTC architecture. Despite having fewer parameters than all baselines, the proposed model achieves the highest detection accuracy of 99.96%, confirming that the additional computational capacity in larger models such as CNN-LSTM (29,456 parameters) does not translate into proportional accuracy gains. The average inference time of 1.091 ms per sample and 6.522 ms per batch of 256 samples confirms that the proposed model is capable of processing network traffic at speeds suitable for real-time detection. The proposed model is faster than LSTM, GRU, and CNN-LSTM, and only marginally slower than MLP, which lacks any temporal processing capability. These results demonstrate that the proposed LNN model achieves the best trade-off between detection accuracy, adversarial robustness, and computational efficiency among all evaluated architectures, making it a practical and deployable solution for real-time DDoS detection in SDN environments and moderately resourced IoT gateways.

**Table 7:** Comparison of existing DDoS detection studies and LNN-based works with the proposed model.

Studies	Year	Techniques	Domain	Temporal adaptation	Accuracy	Adversarial attacks evaluation
^25^	2020	RNN-LSTM	SDN DDoS Detection	No	89.63%	No
^35^	2021	KNN, SVM, and GRU	SDN DDoS Detection	No	99%	No
^1^	2023	LSTM	SDN DDoS Detection	No	96%	No
^49^	2024	DT, SVM, and NB	IoT and SDN DDoS Detection	No	98.2%	No
^11^	2024	SVM, LR, DT, KNN	SDN DDoS Detection	No	99%	No
^33^	2025	DT and CNN	IoT DDoS Detection	No	99%	No
^52^	2026	CNN	SDN DDoS Detection	No	99%	No
^53^	2026	PCA-SVM	IoT DDoS Detection	No	99%	No
Proposed Model	2026	LNNs	IoT and SDN DDoS Detection	Yes	99.96%	Yes

Despite of all of these potential benefits the proposed model may have some challenges during deployment in the IoT and SDN environments. Most of the IoT devices have very much little computation power and memory so the model needs to be deployed carefully and even the edge of such network to avoid any performance issue. DDoS detection depends on fast decision making and LNNs adapt over time, but they must be tuned and trained carefully. LNNs handle temporal variation very well but it still needs a plan for continuous validation. The model can itself be the target of attackers so careful collection of training data is required to avoid poisoned data. SDNs do not have the issue of computation power or memory but deployment in SDN environments also required careful operations during deployments. The integration of SDN and IoT networks are also there so in such environment the deployment can also be challenging.

**Table 8:** Existing applications of liquid neural networks (non-security domains).

Studies	Year	Techniques	Domain	Temporal adaptation
^20^	2023	LNN	Robotics/navigation	Yes
^41^	2024	LTC-GNN	Multi-agent control	Yes
^42^	2024	LTC-AE	Time-series anomaly detection	Yes
^21^	2024	LNN	IoT device classification	Yes
^43^	2025	LTC/CFC	Vehicular misbehavior (IoV)	Yes

**Table 9:** Computational complexity and inference time comparison.

Model	Parameters	Inference time (ms/sample)	Inference time (ms/batch)
MLP	11,994	0.5	3.2
LSTM	16,619	1.4	13.3
GRU	14,576	1.3	8.7
CNN-LSTM	29,456	3.1	19.4
Proposed Model	11,498	1.091	6.522

While the proposed model demonstrates strong detection performance and computational efficiency, several practical limitations must be acknowledged. Although the model contains only 11,498 parameters and achieves an inference time of 1.091 ms per sample on a standard desktop environment, real deployment on severely constrained IoT devices, such as microcontrollers or low-power sensors, may face memory overhead challenges, as even compact PyTorch models require runtime memory for hidden states, gradient buffers, and the ODE solver during continuous-time integration. Latency may also be a concern in devices operating under strict real-time constraints, where sub-millisecond response is required. Furthermore, the LNN’s continuous-time recurrent dynamics, while beneficial for temporal adaptation, add integration complexity compared to simpler feedforward models. Deployment in heterogeneous IoT environments would likely require model compression techniques such as quantization or pruning, and hardware-specific optimizations, which are identified as a direction for future work.

## Conclusion

This research article presented a LNN based model for DDoS detection in IoT and SDN network environments. The proposed model achieved a maximum accuracy of 99.96% on the CICIoT2023 dataset with an FPR of 0.07% and FNR of 0.02%, consistently outperforming the baseline models across all three datasets. Under adversarial conditions generated using the MIMO-MIM framework, which applies momentum-based iterative perturbations to simulate realistic evasion attempts, the proposed model retained accuracy above 90% with a maximum drop of 8.82%, compared to drops ranging from 12.46% to 21.41% across all baseline models. LNN’s adaptive state representation enables it to deal with temporal dependencies and nonlinear relationships in network data and traffic. The model’s ability to adapt its internal dynamics allowed it to track sudden changes more effectively than the existing methods and approaches. This showed that flexible temporal modeling is more important for DDoS detection than traditional stacking deeper architectures of DL models. The results showed that an adaptive state representation helps the model stay stable when traffic conditions vary over time. The ability of proposed model to maintain stable predictions under varying conditions suggests strong potential for deployment in practical environments including IoT and SDN. The proposed model successfully stood stable and proved its generalization and robustness capability when evaluated against adversarial attacks using a MIMO based adversarial framework. The consistent performance across three heterogeneous datasets including SNT-SDN2025, CICIoT2023, and IoT23, with accuracy above 99.43% in all cases confirms the generalization capability of the proposed model. The proposed model further demonstrated the best computational efficiency among all temporal models, with only 11,498 parameters and an inference time of 1.091 ms per sample, confirming its suitability for real-time deployment in resource-aware IoT and SDN environments. An ablation study further confirmed that each architectural component, including the stacked LTC layers, multi-head attention mechanism, batch normalization, and dropout regularization, contributes meaningfully to the model’s detection accuracy and generalization capability. Although the evaluation covered several recent datasets, real deployments may introduce additional challenges such as hardware constraints. Overall, the study highlighted the value of adaptive temporal modeling for improving DDoS detection under adversarial conditions in modern and dynamic network systems.

## Future directions

In future this study will be extended to further optimize the proposed model using model compression and hardware adaptations. Furthermore, the proposed model will be tested in real SDN controllers and IoT devices. The proposed model will also be benchmarked against transformer-based intrusion detection approaches under matched experimental conditions, including adversarial evaluation, given the architectural relevance of self-attention to the proposed design. These investigations will further validate the scalability, practicality, adaptability, and competitiveness of the proposed approach in large-scale, real-world scenarios.

## Data Availability

The datasets used during current study available from corresponding author on reasonable request.

## References

[1] Yungaicela-Naula, N. M., Vargas-Rosales, C., Perez-Diaz, J. A., Jacob, E. & Martinez-Cagnazzo, C. Physical Assessment of an SDN-Based Security Framework for DDoS Attack Mitigation: Introducing the SDN-SlowRate-DDoS Dataset. *IEEE Access.* **11**, 46820–46831 (2023).

[2] Assadhan, B., Bashaiwth, A. & Binsalleeh, H. Enhancing Explanation of LSTM-Based DDoS Attack Classification Using SHAP With Pattern Dependency. *IEEE Access.* **12**, 90707–90725 (2024).

[3] Al-Hadhrami, Y. & Hussain, F. K. DDoS attacks in IoT networks: a comprehensive systematic literature review. *World Wide Web*. **24**, 971–1001 (2021).

[4] Bala, B. & Behal, S. AI techniques for IoT-based DDoS attack detection: Taxonomies, comprehensive review and research challenges. *Comput. Sci. Rev.* **52**, 100631 (2024).

[5] Pakmehr, A., Aßmuth, A., Taheri, N. & Ghaffari, A. DDoS attack detection techniques in IoT networks: a survey. *Cluster Comput.* **27**, 14637–14668 (2024).

[6] Kibret Beshah, Y., Lemma Abebe, S. & Mulugeta Melaku, H. Multi-Stage Adversarial Defense for Online DDoS Attack Detection System in IoT. *IEEE Access.* **13**, 72657–72673 (2025).

[7] Saveetha, D., Maragatham, G., Ponnusamy, V. & Zdravković, N. An Integrated Federated Machine Learning and Blockchain Framework With Optimal Miner Selection for Reliable DDOS Attack Detection. *IEEE Access.* **12**, 127903–127915 (2024).

[8] Beshah, Y. K., Abebe, S. L. & Melaku, H. M. Drift adaptive online DDoS attack detection framework for IoT system. *Electronics* **13**, 1004 (2024).

[9] Singh, C. & Jain, A. K. A comprehensive survey on DDoS attacks detection & mitigation in SDN-IoT network. *e-Prime-Advances Electr. Eng. Electron. Energy*. **8**, 100543 (2024).

[10] Singh, C. & Jain, A. K. Defense Strategies Of DDoS Attacks In SDN-IoT Network: A Survey. *Procedia Comput. Sci.* **259**, 809–817 (2025).

[11] Garba, U. H., Toosi, A. N., Pasha, M. F. & Khan, S. SDN-based detection and mitigation of DDoS attacks on smart homes. *Comput. Commun.* **221**, 29–41 (2024).

[12] Belachew, H. M. et al. Design a robust DDoS attack detection and mitigation scheme in SDN-edge-IoT by leveraging machine learning. *IEEE Access* (2025).

[13] Owusu, E. et al. Online network dos/ddos detection: sampling, change point detection, and machine learning methods. *IEEE Commun. Surv. Tutorials* (2024).

[14] Singh, A., Mishra, P., Vinod, P., Gaur, A. & Conti, M. SFC-NIDS: a sustainable and explainable flow filtering based concept drift-driven security approach for network introspection. *Cluster Comput.* **27**, 9757–9782 (2024).

[15] Alashhab, A. A. et al. Enhancing DDoS attack detection and mitigation in SDN using an ensemble online machine learning model. *IEEE access.* **12**, 51630–51649 (2024).

[16] Hirsi, A. et al. Comprehensive analysis of ddos anomaly detection in software-defined networks. *IEEE Access* (2025).

[17] Chen, R. et al. GBDT-IL: incremental learning of gradient boosting decision trees to detect botnets in internet of things. *Sensors* **24**, 2083 (2024). 38610294 10.3390/s24072083 PMC11014079

[18] Wang, X. et al. Robust beamforming with gradient-based liquid neural network. *IEEE Wirel. Commun. Lett* (2024).

[19] Zhang, K. et al. Dynamic Sequential Neighbor Processing: A Liquid Neural Network-Inspired Framework for Enhanced Graph Neural Networks. *Inf Sci. (Ny)* 122452 (2025).

[20] Chahine, M. et al. Robust flight navigation out of distribution with liquid neural networks. *Sci. Robot*. **8**, eadc8892 (2023). 37075102 10.1126/scirobotics.adc8892

[21] Kumar, R., Swarnkar, M., Sharma, N. & Muskan, M. & others. LNNIoT: A Liquid Neural Network-based Bit-level Method for Classifying IoT Devices. in *IEEE International Conference on Advanced Networks and Telecommunications Systems (ANTS)* 1–6 (2024). 1–6 (2024). (2024).

[22] Neto, E. C. P. et al. CICIoT: A real-time dataset and benchmark for large-scale attacks in IoT environment. *Sensors* 23, 5941 (2023). (2023). 10.3390/s23135941 PMC10346235 37447792

[23] Alawadi, A. S. N. T. (Simulated Network Traffic Using Mininet and Ryu) DDoS Detection Dataset. at (2025). 10.17632/9hz6f62gtk.1

[24] Garcia, S., Parmisano, A. & Erquiaga, M. J. IoT-23: A labeled dataset with malicious and benign IoT network traffic. at (2020). 10.5281/zenodo.4743746

[25] Gadze, J. D., Bamfo-Asante, A. A., Agyemang, J. O., Nunoo-Mensah, H. & Opare, K. A.-B. An investigation into the application of deep learning in the detection and mitigation of DDOS attack on SDN controllers. *Technologies* **9**, 14 (2021).

[26] Pillai, S. E. V. S. & Polimetla, K. Mitigating ddos attacks using sdn-based network security measures. in *International Conference on Integrated Circuits and Communication Systems (ICICACS)* 1–7 (2024). 1–7 (2024). (2024).

[27] Wang, K., Fu, Y., Duan, X. & Liu, T. Detection and mitigation of DDoS attacks based on multi-dimensional characteristics in SDN. *Sci. Rep.* **14**, 16421 (2024). 39014041 10.1038/s41598-024-66907-z PMC11253008

[28] Alhijawi, B., Almajali, S., Elgala, H., Salameh, H. B. & Ayyash, M. A survey on DoS/DDoS mitigation techniques in SDNs: Classification, comparison, solutions, testing tools and datasets. *Comput. Electr. Eng.* **99**, 107706 (2022).

[29] Ran, L. et al. Defending saturation attacks on SDN controller: A confusable instance analysis-based algorithm. *Comput. Networks*. **213**, 109098 (2022).

[30] Ali, M. H. et al. Threat analysis and distributed denial of service (DDoS) attack recognition in the internet of things (IoT). *Electronics* **11**, 494 (2022).

[31] Alahmadi, A. A. et al. DDoS attack detection in IoT-based networks using machine learning models: A survey and research directions. *Electronics* **12**, 3103 (2023).

[32] Shukla, P., Krishna, C. R. & Patil, N. V. Iot traffic-based DDoS attacks detection mechanisms: A comprehensive review. *J Supercomput* **80**, (2024).

[33] Fida, M. R., Ahmed, A. H. & Arsalaan, A. S. IoTShield: Defending IoT Systems Against Prevalent Attacks Using Programmable Networks. *IEEE Access.* **13**, 136446–136457 (2025).

[34] Al-Dunainawi, Y., Al-Kaseem, B. R. & Al-Raweshidy, H. S. Optimized Artificial Intelligence Model for DDoS Detection in SDN Environment. *IEEE Access.* **11**, 106733–106748 (2023).

[35] Yungaicela-Naula, N. M., Vargas-Rosales, C. & Perez-Diaz, J. A. SDN-Based Architecture for Transport and Application Layer DDoS Attack Detection by Using Machine and Deep Learning. *IEEE Access.* **9**, 108495–108512 (2021).

[36] Lent, D. M. B. et al. An unsupervised generative adversarial network system to detect ddos attacks in sdn. *IEEE Access.* **12**, 70690–70706 (2024).

[37] Alasmary, F., Alraddadi, S., Al-Ahmadi, S. & Al-Muhtadi, J. ShieldRNN: A Distributed Flow-Based DDoS Detection Solution for IoT Using Sequence Majority Voting. *IEEE Access.* **10**, 88263–88275 (2022).

[38] Gleeson, P., Lung, D., Grosu, R., Hasani, R. & Larson, S. D. c302: a multiscale framework for modelling the nervous system of Caenorhabditis elegans. *Philos. Trans. R Soc. B Biol. Sci.* **373**, 20170379 (2018). 10.1098/rstb.2017.0379 PMC6158223 30201842

[39] Hasani, R., Lechner, M., Amini, A., Rus, D. & Grosu, R. Liquid time-constant networks. in *Proceedings of the AAAI Conference on Artificial Intelligence* vol. 35 7657–7666 (2021).

[40] Hasani, R. et al. Closed-form continuous-time neural networks. *Nat. Mach. Intell.* **4**, 992–1003 (2022).

[41] Marino, A., Pacchierotti, C. & Giordano, P. R. Liquid-graph time-constant network for multi-agent systems control. in *IEEE 63rd Conference on Decision and Control (CDC)* 793–800 (2024). 793–800 (2024). (2024).

[42] Sailesh, R. S., Preethan, J. & Sivaprakash, S. & others. Ltc-ae: Liquid time constant autoencoders for time series anomaly detection. in *IEEE International Conference on Information Technology, Electronics and Intelligent Communication Systems (ICITEICS)* 1–6 (2024). 1–6 (2024). (2024).

[43] Gong, Y. & Hu, B. J. Lightweight framework for misbehavior detection in internet of vehicles. *J. Highw Transp. Res. Dev. (English Ed.* **19**, 13–17 (2025).

[44] Polat, O. et al. Supervised and deep learning techniques for DDoS detection in software-defined network architectures: a systematic review. *Eng. Sci. Technol. Int. J.* **75**, 102290 (2026).

[45] Patro, P., Kumar, K., Kumar, G. S. & Sahu, A. K. Intelligent data classification using optimized fuzzy neural network and improved cuckoo search optimization. *Iran. J. Fuzzy Syst.* **20**, 155–169 (2023).

[46] Ragunthar, T. et al. Machine Learning-Driven Intrusion Detection Systems for Anomaly Detection in Vehicular Ad-Hoc Networks. *Trans. Emerg. Telecommun Technol.* **37**, e70363 (2026).

[47] Patro, P., Kumar, K., Kumar, G. S. & Swain, G. Similarity and wavelet transform based data partitioning and parameter learning for fuzzy neural network. *J. King Saud Univ. Inf. Sci.* **34**, 3424–3432 (2022).

[48] Mallikarjun, A. & Patro, P. aHybrid Data Balancing with MLP Probabilities-based Categorical Boosting Model for Robust Intrusion Detection System in IoT Environment. *Syst Soft Comput* 200443 (2026).

[49] Jyothsna, V. & others. Defending against IoT threats: A comprehensive framework with advanced models and real-time threat intelligence for DDoS detection. in *2nd International Conference on Networking and Communications (ICNWC)* 1–7 (2024). 1–7 (2024). (2024).

[50] Jain, M., Sahoo, A. & Singh, P. An Empirical Study of the Concept Drift Driven Machine Learning for IoT Network Anomaly Detection. in Network Optimization in Intelligent Internet of Things Applications 227–242 (Chapman and Hall/CRC, (2024).

[51] Clinton, U. B. & Hoque, N. Robindro Singh, K. Classification of DDoS attack traffic on SDN network environment using deep learning. *Cybersecurity* **7**, 23 (2024).

[52] Pham-Thai, B., Nguyen-Le-Ha, M. & Nguyen-Khanh, T. Le-Trung, Q. An Approach to Attack Classification for Programmable Network Infrastructure Using Machine Learning and Deep Learning Solutions. *IEEE Access.* **14**, 4886–4916 (2026).

[53] Tajudeen, K. O., Ameen, A. O. & Adeniyi, A. E. Hybrid deep learning models for detecting user datagram protocol-based distributed denial of service attacks in internet of things networks. *Discov Internet Things* (2026).

